# High Cell Density Cultivation of HEK293 Cells Using a 2L Membrane‐Stirred Bioreactor

**DOI:** 10.1002/bit.70317

**Published:** 2026-07-26

**Authors:** Lennart Jacobtorweihe, Yasemin van Heuvel, Kathrina Scheiermann, Patrick Bongartz, Yvonne Genzel, Udo Reichl

**Affiliations:** ^1^ Max Planck Institute for Dynamics of Complex Technical Systems Magdeburg Germany; ^2^ BioThrust GmbH Aachen Germany; ^3^ Otto‐von‐Guericke‐University Magdeburg Germany

**Keywords:** bioreactor, ComfyCell, foam free, HEK293 suspension cells, high‐cell‐density

## Abstract

The use of perfusion systems for the continuous cultivation of animal cells at high cell density (HCD) for the production of recombinant proteins, viruses, and viral vectors has many advantages. Besides an enhancement of volumetric productivity and high cell viability, the product quality can be improved through a steady‐state environment. However, the establishment of such a process intensification method using stirred tank bioreactors (STRs) for cultivations at very high cell concentrations (> 50 × 10^6^ cells mL^−1^) can be challenging. In particular, limitations in the oxygen supply, CO_2_ stripping, high viscosity, and excessive foam formation can impede cell viability and product yields. In this study, we investigated a novel membrane‐stirrer (MemStir) based bioreactor that employs multiple hollow‐fiber membrane sheets arranged in a device that facilitates efficient gas transfer through bubble‐free diffusion for HCD cultivations. We found that the oxygen transfer rates at different tip speeds and gas flow rates resulted in volumetric mass transfer coefficient (kLa) values of 10–30 h^−1^ enabling sufficient oxygen supply to support concentrations exceeding 50 × 10^6^ cells mL^−1^. The functionality of the membrane‐stirrer was benchmarked in batch mode against shake flasks and a conventional STR equipped with a pitched‐blade impeller and an l‐drilled hole sparger. Suspension growth of a human embryonic kidney 293 LTV (HEK‐LTV) cell in the MemStir system was comparable to the cell growth in a STR. Using pure oxygen for aeration, a maximum viable cell concentration of 7.6 × 10^6^ cells mL^−1^ was achieved. As a starting point for further optimization, we investigated the impact of the MemStir system on viable cell concentration, cell viability, dissolved oxygen partial pressure, pH control and foam formation in HCD cultivations in perfusion mode. Using the MemStir system equipped with an alternating tangential flow system, HEK‐LTV cells reached concentrations of up to 92 × 10^6^ cells mL^−1^ with a specific growth rate of 0.022 h^−1^ in perfusion mode. Moreover, controlling the pH in the range 7.0 to 7.3 was easily achieved, and no foam formation was observed. Overall, these results suggest that the MemStir system is a viable option for HCD cultivation because it can overcome limitations of both aeration and foam formation.

**Abbreviations**
ATF: alternating tangential flow filtration
CHO: Chinese hamster ovary
CSPR: cell‐specific perfusion rate
DO: dissolved oxygen concentration
EBNA: Epstein‐Barr Nuclear Antigene 1
HCD: high‐cell‐density
HEK293: human embryonic kidney 293
k_L_a: volumetric mass transfer coefficient
MemStir: membrane‐stirrer
OTR: oxygen transfer rate
rpm: rounds per minute
RV: reactor volumes
STR: stirred tank reactor
VCC: viable cell concentration
vvm: vessel volumes per minute

## 1 Introduction

Chinese hamster ovary (CHO) cell lines are the preferred host for producing recombinant proteins for biomedical use, such as antibodies, which make up 80% of approved protein‐based drugs (Zhang et al. 2024). Because they lack human‐like glycan patterns, cell lines such as human embryonic kidney 293 (HEK293) cells are considered as an alternative for the production of biopharmaceuticals (Zhang et al. 2024; Pulix et al. 2021). HEK293 cells are derived from fetal kidney tissue and grow easily in adherent and suspension cultures with high growth rates (Thomas and Smart 2005; Tan et al. 2021). This makes them ideal host cells for research at small scales and manufacturing at large scales. Like other human cells, HEK293 cells can perform essential post‐translational modifications, such as glycosylation and proteolytic processing, to ensure the activity and stability of recombinant proteins (Tan et al. 2021; Durocher and Butler 2009). HEK293 derivatives, such as 293 T cells and cell lines adapted to serum‐free media (e.g., HEK293F cells), further enhance this capability by improving expression capacity and better cell growth, thereby simplifying bioprocesses (Zhang et al. 2024; Smesnik et al. 2026). HEK293 cells support transient and stable expression strategies for producing recombinant proteins, enabling rapid prototyping and the sustained manufacturing of complex biologics, such as antibodies, enzymes and membrane proteins. They are also central for producing viruses, viral vectors, and virus‐like particles for research and gene therapy, including adeno‐associated virus, vesicular stomatitis virus, lentiviral and adenoviral vectors (Pulix et al. 2021; Tan et al. 2021; Hamusics et al. 2025). In this context, HEK293‐based producer systems can even complement necessary helper functions, such as E1 A/B proteins, which are required to assemble vector genomes into functional vector particles under controlled conditions (Tan et al. 2021; Wold and Toth 2013). Ongoing innovations aim to increase product yields, improve product consistency, and reduce costs. For example, by increasing volumetric productivity in fed‐batch high‐cell‐density (HCD) cultivations and perfusion cultures (Shen et al. 2024; Silva et al. 2025; Hein et al. 2023; Tran and Kamen 2022).

HCD cultivation of mammalian cells is fundamental to modern and sustainable biopharmaceutical manufacturing. It enables a smaller process footprint and increased productivity when manufacturing monoclonal antibodies, vaccines, and other biologics (Pelz 2022; Drobnjakovic et al. 2023). HCD processes depend on an optimized media composition and feeding strategies, ranging from intensified fed‐batch to continuous perfusion with cell‐retention devices such as inclined or acoustic settlers, as well as membrane‐based filtration devices, including alternating tangential flow filtration (ATF), tangential flow filtration (TFF), and tangential flow depth filtration (TFDF)) (Jacobtorweihe et al. 2025; Göbel 2022; Robitaille 2024). However, engineering limitations regarding oxygen transfer, CO_2_ stripping, mixing, shear and foam formation become increasingly restrictive at scale (Karimi Alavijeh et al. 2022; de Mello et al. 2023; von Werz et al. 2026). This necessitates a tailored bioreactor design and adequate gas‐liquid mass transfer strategies (von Werz et al. 2026). Because the cellular physiology is shaped by nutrient gradients and by‐product accumulation (e.g., lactate and ammonia), therefore precise control of pH values, osmolality and temperature is necessary to ensure high cell viability and low cellular stress, and to maintain product quality (Xu et al. 2018; Schneider 1996).

In this study, we performed HCD cultivations using a bioreactor (IKA habitat 2 L) equipped with a membrane‐stirrer (MemStir) system (BioThrust GmbH, Germany) as an alternative to a conventional stirred tank reactor (STR) with a sparger and pitched‐blade impeller. The MemStir system enables efficient gas exchange, based on diffusion through hollow‐fiber membranes. It allows for bubble‐free aeration and efficient CO_2_ stripping (Bongartz et al. 2023; 2021a). We evaluated the MemStir system for HEK293 LTV (HEK‐LTV) cell cultivation in batch and perfusion modes, benchmarking the results against those of STR and shake flask cultivations. First, we investigated the volumetric mass transfer coefficients (k_L_a) and oxygen transfer rates (OTR) of the MemStir system under different stirring speeds and working volumes to identify maximum cell concentrations that can be achieved. Then, the performance of batch cultivations of HEK‐LTV cells in shake flasks and conventional STR was benchmarked against the IKA habitat bioreactor, equipped with the MemStir system. Finally, the overall potential of the MemStir system was evaluated in two HCD cultivations in perfusion mode.

## 2 Materials and Methods

### 2.1 Cell Culture and Analytics

Adherent HEK‐LTV cells (Nordic Biosite, Sweden), adapted to suspension growth by BioInvent International AB, were routinely passaged in Dynamis medium (Gibco, Thermo Fisher Scientific, USA) and seeded at 0.8 × 10^6^ cells/mL, twice per week. Cells were cultured in 125 mL non‐baffled shake flasks at 37°C, 5% CO_2_ and 150 rounds per minute (rpm) (50 mm throw). Cell viability and viable cell concentration (VCC) were measured using a ViCell XR (Beckman Coulter, USA). For cell concentrations exceeding 10 × 10^6^ cells/mL, the cells were diluted in 1x porcine trypsin (Thermo Fisher Scientific, USA) and incubated for 10 min at 37°C to reduce the formation of cell aggregates prior to counting. For the determination of glucose, glutamine, glutamate, ammonium and lactate concentrations, a Cedex® metabolite analyzer (Roche Holding AG, Switzerland) was used.

### 2.2 Bioreactor Cultivations

The cells were cultured in a 2 L IKA habitat cell system (IKA‐Werke GmbH & Co. KG, Germany) using a MemStir system for gassing and stirring with a fluid wave coupling element (Figure 1, BioThrust GmbH, Germany). Cells were stirred at 37°C, 60 rpm and pH was set to 7.2 controlled by CO_2_ and NaHCO_3_ with a dead band of 0.05. The dissolved oxygen concentration (DO) was set to 50%, calibrated with air and controlled with a flow ranging from 400 to 2000 cm^3^/min (0.65 vessel volumes per minute (vvm) to 3.3 vvm) using N_2_, air and O_2_. The proportions were adjusted to control the DO (Table S1).

**Figure 1:**
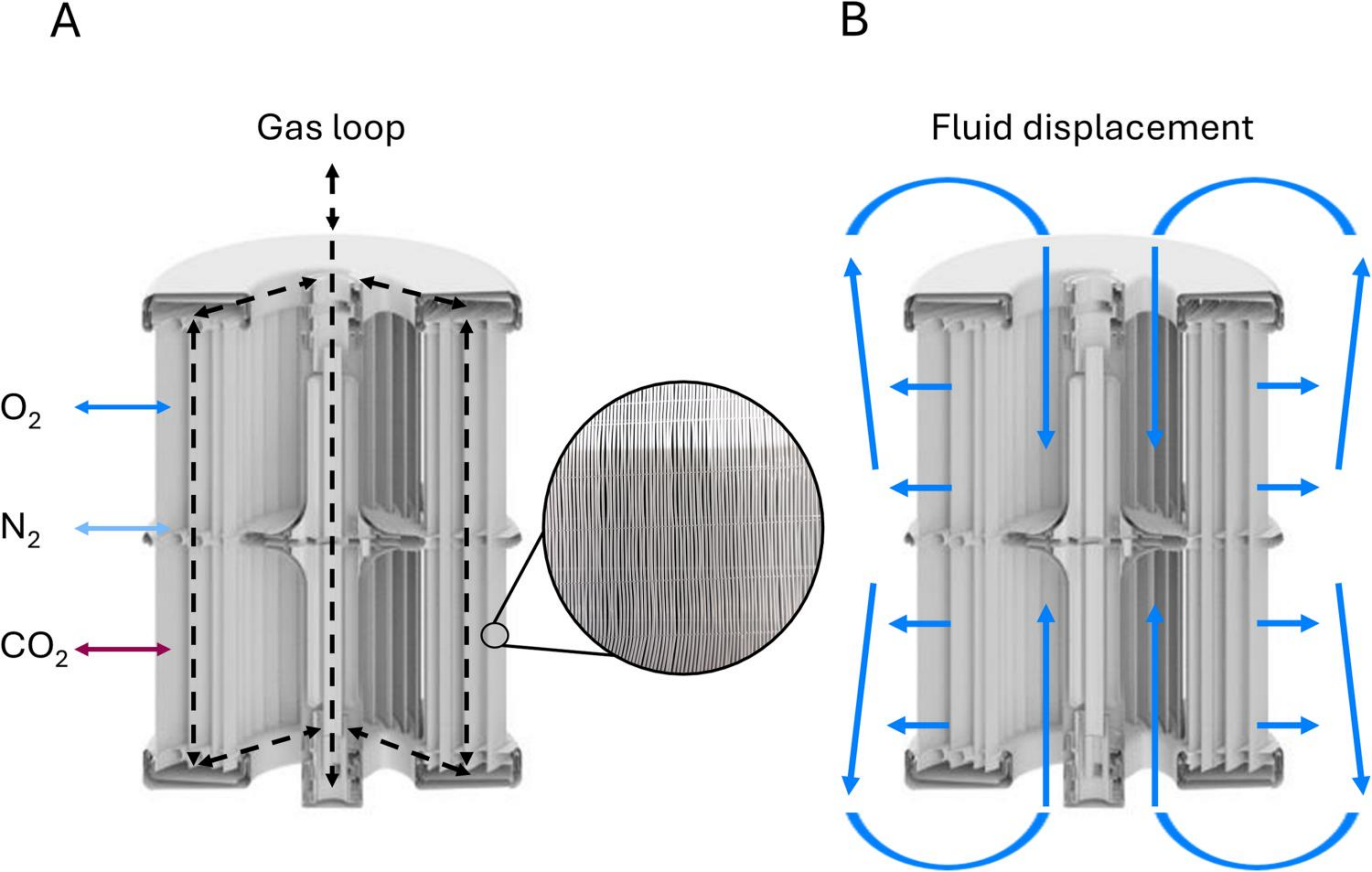
The BioThrust membrane‐stirrer technology. A: Gas loop direction (black dotted arrows) and bidirectional gas exchange via the membrane surface (colored arrows, e.g., O_2_, N_2_ and CO_2_); the inset shows a zoom of the hollow‐fiber sheet. B: Fluid displacement direction through the membrane‐stirrer (blue arrows).

A 2 L DASGIP STR (Eppendorf SE, Germany) was used to stir cells at 150 rpm with a working volume of 0.3 L. DO was set to 50% and controlled via an l‐drilled hole sparger and a gas flow of 50–150 cm^3^/min (0.13–0.375 vvm). The pH was controlled as described in the IKA habitat system. In batch mode, the cells were cultured in 400 mL (DASGIP reactor) and 600 mL (BioThrust reactor) Dynamis medium and seeded at 0.8 × 10^6^ cells/mL.

### 2.3 Membrane‐Stirrer Technology

The membrane‐stirrer consists of many dense, gas permeable hollow‐fiber membranes arranged in a mesh pattern. This enables an efficient bubble‐free gas transfer through a large effective membrane surface area (1917 cm^2^). Process gases pass bi‐directionally through the dense hollow fibres into the surrounding liquid via molecular diffusion (Figure 1A); O_2_, CO_2_ and N_2_ can diffuse out and back in (Bongartz 2021b). In addition, the vertical bi‐circular flow pattern ensures efficient homogenous mixing by drawing liquid from the top and bottom of the membrane‐stirrer and releasing it radially outward (Figure 1B).

### 2.4 Perfusion Mode

An ATF2 system (Refine Technology, USA) was used as cell retention device with a 980 cm^2^ PES hollow‐fibre membrane and a pore size of 0.2 µm (Spectrum Labs, USA) (Figure 2). The circulation rate was set to 0.8 to 1.1 L/min, depending on the VCC. Dynamis medium containing 1 U/mL TrypLE (Gibco, Thermo Fisher Scientific, USA) to reduce cell aggregates was fed at a cell‐specific perfusion rate (CSPR) of 48 pL/(cell×day). The perfusion rate was manually adjusted every 12 to 24 h, and the feed and harvest were controlled via a double‐head peristaltic pump (Figure 2). In contrast to batch cultivations, a working volume of 900 mL was used to increase the membrane to working volume ratio. DO was controlled at 50% under the same conditions as in batch mode; no further gas input was necessary.

**Figure 2:**
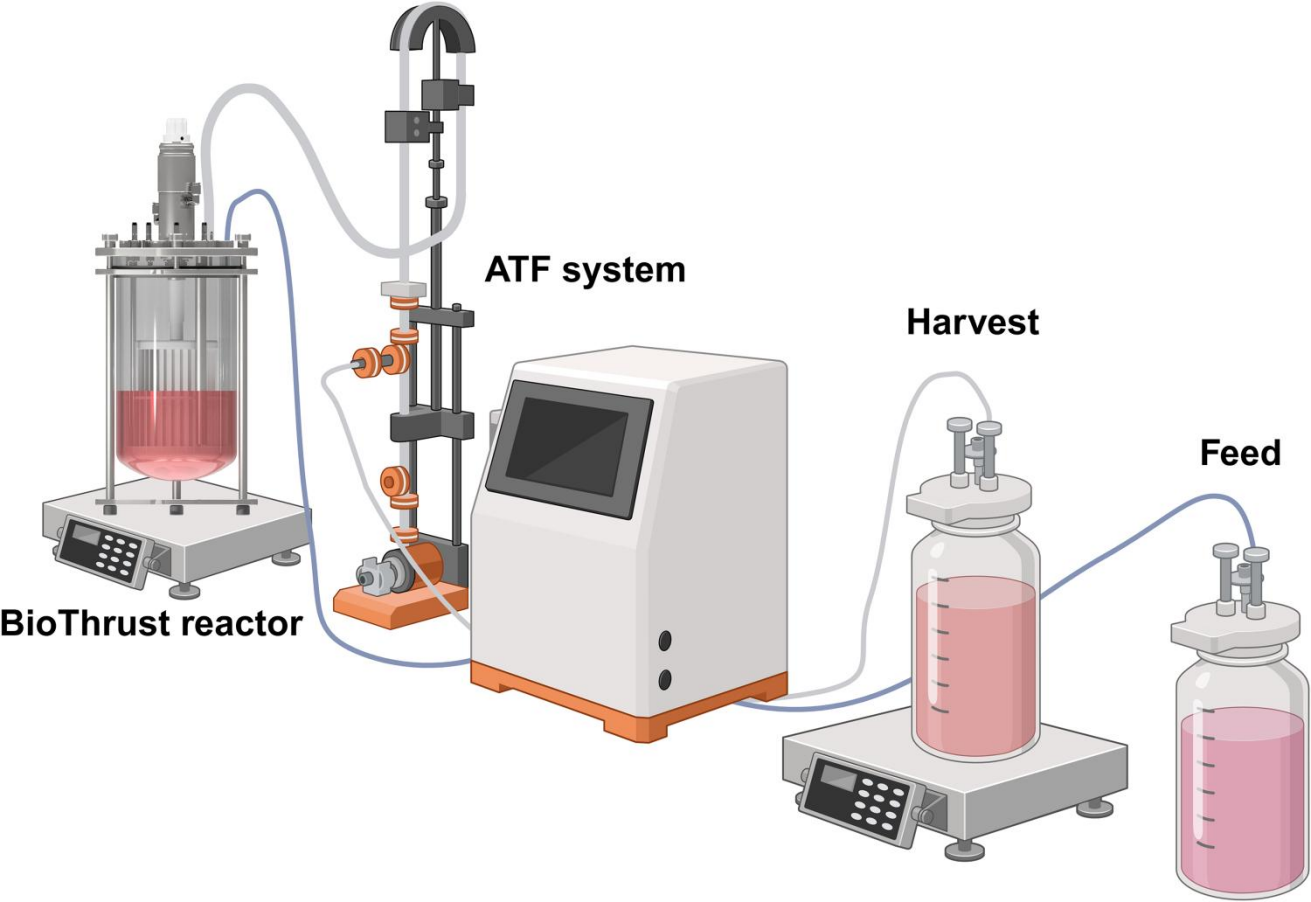
Perfusion setup using a 2 L glass bioreactor with the membrane‐stirrer system and an ATF system for cell retention (cut‐off 0.2 µm). The feed and harvest rates were set according to a cell‐specific perfusion rate and adjusted every 12 to 24 h (created with biorender. com).

### 2.5 Determination of the k_L_a Value for the Memstir System

The k_L_a was determined using the gassing‐out method described in detail by the DECHEMA guideline (Bauer et al. 2020). Therefore, DO was alternately depleted and replenished in a 1× PBS buffer at pH 7.2 within the bioreactor at 37°C and typical stirring speeds from 20 to 200 rpm. Oxygen (introduced with air) was removed by a nitrogen gas flow and reintroduced using air.

DO concentrations were measured using an optical oxygen sensor (PyroScience GmbH, Germany) at a rate of 1 s. The sensor was connected to an external computer using the pyro workbench software (PyroScience GmbH, Germany). After pH and DO sensors were calibrated and stabilized at 37°C, measurements were initiated under agitation. Each cultivation continued until a DO saturation of 100% was reached. For the k_L_a determination, DO ranges between 10% and 90% saturation were evaluated. Stirrer speeds were varied from 20 to 200 rpm, and all experiments were performed in triplicate. The OTR and k_L_a values were calculated according to Equations 1 and 2.

(1) $$\frac{\mathrm{dC}}{\mathrm{dt}}=k_{L}a\times \left(C_{O2}^{*}-C\right)$$

where C (mmol/L) is the dissolved gas concentration, in this case O_2_, and $C_{O2}^{*}$ the equilibrium concentration at the phase boundary (mmol/L).

(2) $$\mathrm{OTR}=k_{L}a\;\times \left(C_{O2}^{*}-C_{O2}\right)$$

With the oxygen transfer rate (*OTR*, mmol/L/h) and $C_{O2}$ the actual liquid concentration (mmol/L).

### 2.6 Data Evaluation

The experimental data were exported as comma‐separated values (CSV) files and imported into Microsoft Excel (Microsoft Corp., USA) for analysis and visualization. Graphs were generated using OriginPro (ADDITIVE Soft‐ und Hardware für Technik und Wissenschaft GmbH, Germany). Bioreactor visualizations were made using Autodesk Inventor Professional (Version 2024) (Autodesk, Inc., USA) and biorender.com (BioRender Science Suite Inc., Canada).

## 3 Results and Discussion

To evaluate the MemStir system for HCD cultivation of HEK‐LTV cells, we first determined the k_L_a values under various process conditions. Additionally, we compared batch cultivations using a conventional STR, a bioreactor equipped with a MemStir system, and shake flasks regarding the growth characteristics of HEK‐LTV cells. Finally, HCD cultivations were performed in perfusion mode using the MemStir system and an ATF system as the cell retention device.

### 3.1 Determination of K_L_a Values

The effect of stirrer speed and aeration rate on oxygen transfer was evaluated using the MemStir system with a 1 L working volume of PBS using oxygen (introduced with air). Therefore, we determined the k_L_a values and the OTR over a range of operating conditions (Figure 3A,B).

**Figure 3:**
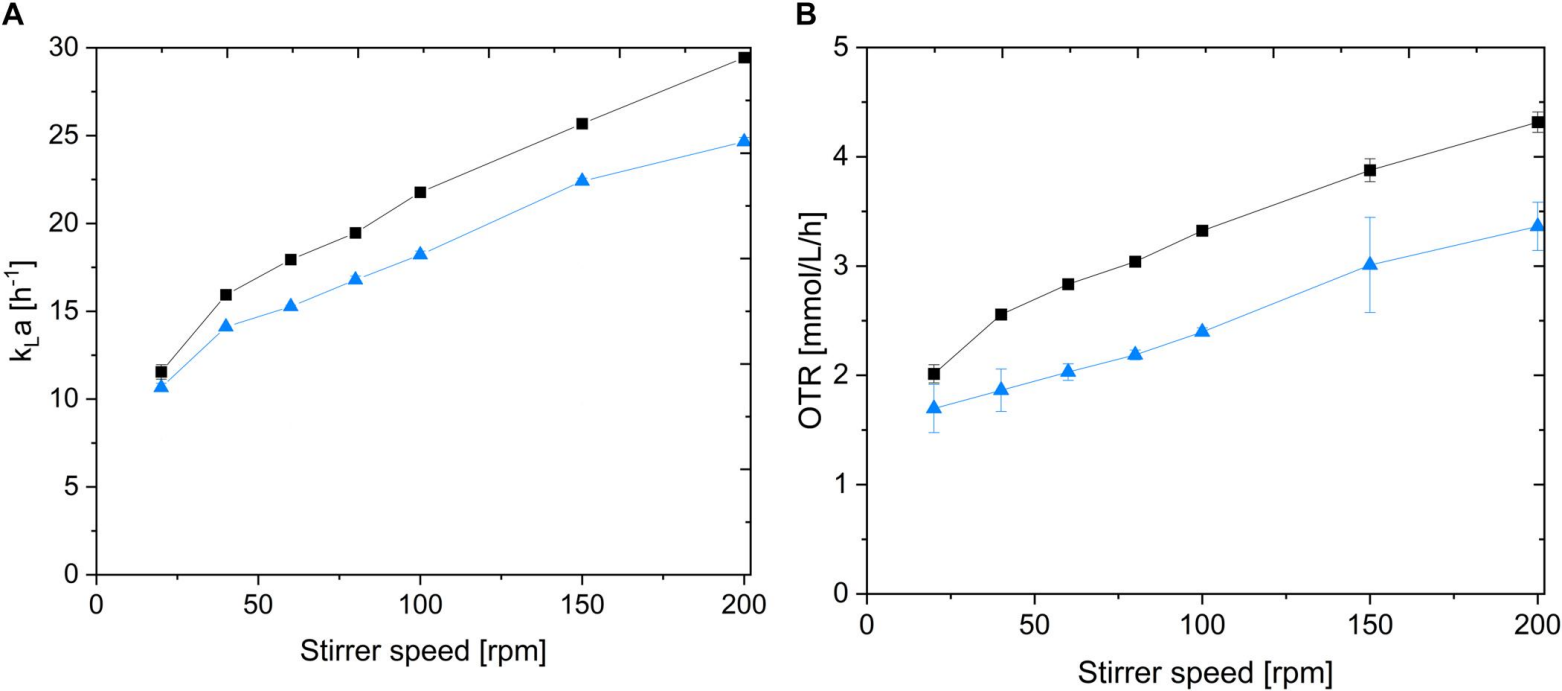
Oxygen transfer in a 2 L IKA bioreactor equipped with a membrane‐stirred system at 20–200 rpm and 1 L working volume (PBS). Aeration rates: 1 vvm (2000 mL min^−1^, ▲) and 2 vvm (4000 mL min^−1^, ■). A Volumetric mass transfer coefficient (k_L_a, h^−1^), B oxygen transfer rate (OTR, mmol L^−1^ h^−1^), both as a function of stirrer speed. Mean and standard deviation of technical triplicates.

As shown in Figure 3A, the k_L_a increased with increasing stirrer speed at both tested aeration rates (1 and 2 vvm). This increase was more pronounced at higher gas flow rates. At 2 vvm, the k_L_a value increased sharply from approximately 12 h^−1^ at 20 rpm to nearly 30 h^−1^ at the highest stirrer speed (200 rpm), indicating a strong synergistic effect between agitation and aeration. At 1 vvm, the k_L_a values also increased with stirrer speed, but had lower absolute values and reduced slopes compared to 2 vvm. Nevertheless, the highest k_L_a achieved was 29.5 h^−1^.

Consistent with the k_L_a trends, the OTR increased with stirrer speed and aeration rate (Figure 3B). At 2 vvm, OTR values increased nearly linearly across the investigated stirrer speed range, reaching values above 4 mmol × L^−1^ × h^−1^ at the highest speed. At 1 vvm, the OTR gradually increased with agitation but remained consistently lower (maximum 3.3 mmol × L^−1^ × h^−1^).

Overall, both k_L_a and OTR increased strongly with stirrer speed. These results demonstrate that efficient oxygen transfer requires the combined contribution of sufficient gas flow and homogenous agitation.

Further experiments were performed (Supporting Figure S1) to ensure sufficient aeration under the conditions used for the perfusion process (working volumes of 0.6 , 0.9 and 1.4 L). However, the ATF system was not turned on for this determination. In previous studies, only a minor to non‐significant effect of the ATF system on the k_L_a value was determined (Karst 2016; Soice 2017). Here, all k_L_a values exceeded 10 h^−1^. Ozturk estimated k_L_a values within the range of 5–55 h^−1^ to allow for the cultivation of up to 10^8^ mammalian cells/mL in perfusion mode (Ozturk 1996). Based on Ozturk's assumptions and an average cell‐specific oxygen uptake rate of 1.58 × 10^−13 ^mol/(cells × h) reported for HEK suspension cells (Martínez‐Monge et al. 2018), maximum concentrations of 4.9 × 10^7^ cells/mL and 1.5 × 10^8^ cells/mL could be achieved for k_L_a values of 10 and 30 h^−1^, respectively.

The evaluated k_L_a data however can be considered an underestimation for the MemStir system. Although the k_L_a was measured as done in STRs with ring or micro sparger (including bubble size dependency, bubble flow regimes, hydrodynamics, etc.), this does not explicitly resolve the interfacial area of gas exchange, which is of main importance for membrane aerated bioreactors (Côté et al. 1989; Berth et al. 2019).

### 3.2 Culturing HEK‐LTV Cells in Different Bioreactor Systems

To evaluate the MemStir system, HEK‐LTV cells were grown in batch mode and compared with cells grown in shake flasks and STRs. In all three culture systems, the HEK‐LTV cells exhibited exponential growth until 96 h post‐inoculation. The maximum cell‐specific growth rate (µ_max_) of cells cultivated in the STRs was lower between 24 and 72 h (0.014 h^−1^) compared to the shake flask (0.021, 0.022, and 0.023 h^−1^) and MemStir systems (0.025 and 0.028 h^−1^) but sharply increased between 72 and 96 h (0.053 h^−1^). At low concentrations, HEK‐LTV cells showed stronger aggregate formation in the STR until 72 h. Therefore, the VCC measurements taken until approximately 72 h post‐inoculation may have been biased and were similar to those of the shake flask and MemStir system cultures. Finally, the maximum viable cell concentrations were similar for all conditions, reaching 7.2‐ to 7.6 × 10^6^ cells/mL. HEK‐LTV cells grown in shake flasks exhibited a faster reduction in viability between 144 and 168 h. In contrast, viability remained above 85% in both bioreactor systems during that time Figure 4.

**Figure 4:**
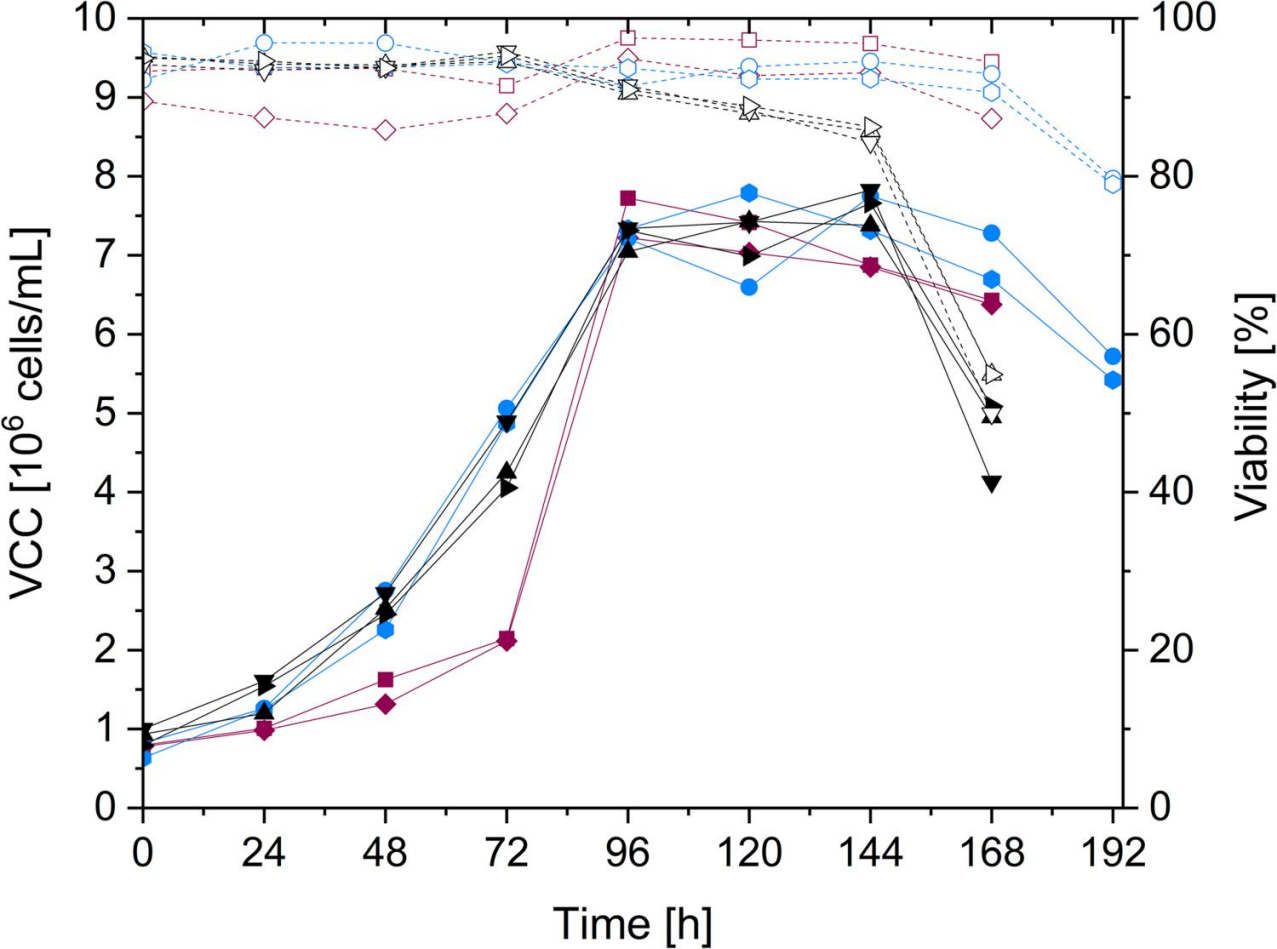
Growth of HEK‐LTV cells in batch mode using shake flasks **(▲ ▼** 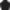 **)**, the MemStir system **(●** ⬟ and a stirred tank reactor **(■ ♦).** Full symbols and lines indicate viable cell concentrations; viability is shown by dashed lines and empty symbols.

The determined growth characteristics were comparable to those of other HEK293 cell lines. HEK‐293T suspension‐adapted cells reached a concentration of 11 × 10^6^ cells/mL 7 days post inoculation, with a *µ*_max_ of 0.034 h^−1^ (Lomba et al. 2021). For cultivating HEK293‐EBNA (Epstein‐Barr Nuclear Antigene 1) in squared shake flasks, a maximum VCC of 5 × 10^6^ cells/mL was observed in batch mode (Muller et al. 2005).

The next step was to evaluate the possibility of conducting HCD cultivations using the MemStir system.

### 3.3 High‐Cell‐Density Cultivations

The production of recombinant proteins and viruses at HCD is an increasingly relevant subject, with the objectives of achieving higher harvest yields, efficiently producing substantial quantities of product, and establishing more sustainable processes. Manufacturing at HCD can be challenging and complex due to increased demands of nutrients and oxygen, as well as the risk of accumulating inhibitors of growth and product formation. Using a micro sparger to facilitate high oxygen levels has been shown to generate substantial foaming through bubble formation (Flynn et al. 2024; Coffman et al. 2021; Druzinec et al. 2015). To overcome this issue, we tested the cultivation of HEK‐LTV cells at HCD (> 50 × 10^6^ cells/mL) using the MemStir system, which allows for direct diffusion of oxygen in the medium. The target DO was set to 50%.

First, HEK‐LTV cells were cultured in perfusion mode using the ATF system, with a targeted CSPR of 40 pL/(cell×day) and a limitation to a maximum perfusion volume of 10 L (Figure 5A and B, CSPR in Supplementary). Perfusion was initiated after 48 h of batch growth and stopped at 197 h. The maximum perfusion rate was set below three reactor volumes per day (RV/day). The HEK‐LTV cells reached a concentration of 62.5 × 10^6^ cells/mL at 202 h post inoculation. The µ_max_ during perfusion (at 48 and 197 h) was 0.020 h^−1^, which was 25% lower than that observed in batch mode (0.034 h^−1^). Our group has previously observed a reduction in the cell‐specific growth rate of HEK293‐F cells when cultured in perfusion mode (Hein et al. 2023). Nutrient concentrations did not reach limiting levels throughout the perfusion process. Glucose concentrations were maintained within the range of 15–20 mM, while lactate concentration peaked during batch growth, reaching up to 17 mM. During perfusion, the target glucose concentration was above 10 mM. After perfusion was terminated, the ammonium concentration increased from below 1 mM up to 4.9 mM. Accumulation of 2–5 mM ammonium and 20–30 mM lactate can negatively impact cell growth, viability and cell‐specific productivity (Schneider 1996; Cruz et al. 2000; Ozturk et al. 1992). Due to the determination of the perfusion mode and a subsequent limitation of the nutrient supply, the overall VCC decreased (Figure 5A). As hypothesized, no oxygen limitation or foam formation was observed for the MemStir system. After 130 h, fluctuations between 20% and 80% DO occurred at the chosen DO cascade setting (Figure S3A). Thus, the settings were modified for the second cultivation to ensure a stable DO. The maximum cell concentration was clearly limited by the stop of perfusion, as well as the constant delivery of nutrients and the removal of metabolic byproducts. Hence, in the second experiment, the total perfusion volume was increased to 28.2 L, to determine whether VCCs of > 65 × 10^6^ cells/mL were possible with this reactor setting.

**Figure 5:**
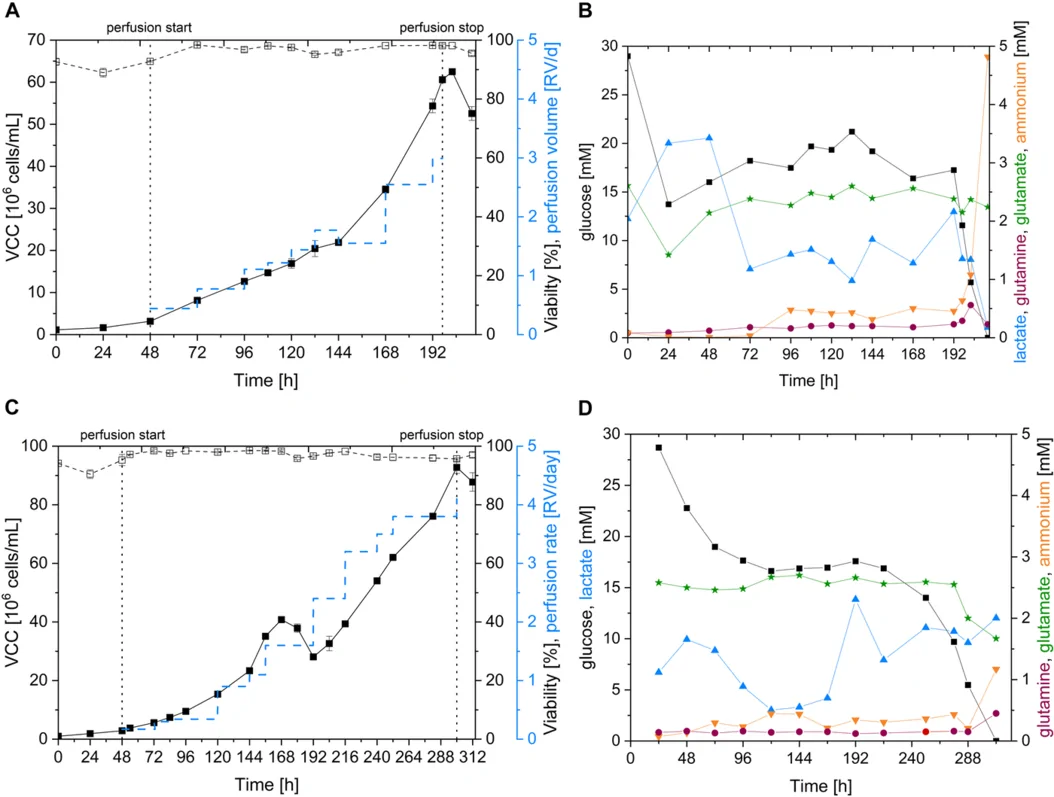
High‐cell‐density cultivation of HEK‐LTV cells in Dynamis medium in the MemStir system using an ATF2 system (0.2 µm cut‐off) for cell retention. (A,B) Cultivation with a fixed maximum total perfusion volume of 10.4 L. (A) VCC (■), viability (□) and perfusion rate (dashed blue line); (B) Concentration of main metabolites. (C,D) Cultivation with a fixed maximum total perfusion volume of 28.2 L. C: VCC (■), viability (□) and perfusion rate (dashed blue line); (D) Concentration of main metabolites. Working volume was increased between 168 and 192 h from 0.9 to 1.4 L to increase membrane surface for improved aeration. VCC, mean and standard deviation of technical duplicates.

During the second cultivation, perfusion was initiated after 48 h of batch growth and ended after 312 h. The working volume was increased from 0.9 to 1.4 L between 168 and 192 h. This increase provided a higher membrane surface‐to‐volume ratio, due to the geometry of the MemStir system, thereby resulting in a higher k_L_a and improved aeration (Figure S1). To further increase the k_L_a, the stirrer speed was increased to 70 rpm after 230 h and 85 rpm after 250 h. A cell‐specific growth rate of 0.022 h^−1^ was observed from the start of perfusion until the increase in working volume. After increasing the total working volume, the cell‐specific growth rate decreased to 0.011 h^−1^ (between 192 and 300 h. Finally, a maximum VCC of 93 × 10^6^ cells/mL was reached. Metabolite measurements indicated sufficient nutrient supply until the perfusion stopped at 300 h post‐inoculation. The lactate concentration peaked at 14 mM after 168 h because the permeate pump was paused to increase the working volume only in the feed. Once the permeate pump was restarted, the lactate concentration decreased. By the end of the perfusion (after 300 h), the glucose level was below 3 mM. The reduction in glutamine concentration and the concurrent accumulation of ammonium suggest the potential use of glutamine as an alternative carbon source and a potential shift in metabolism (Figure 5D) (Lee et al. 2003).

The final perfusion rates of 3 RV/day and 4 RV/day for the first and second cultivations, respectively, were rather high. Typically, perfusion rates below 2 RV/day are the goal for industrial processes (Pollock et al. 2013; Lin et al. 2017). In the future, the perfusion strategy could be improved to reduce medium consumption, making the process more sustainable and economical. However, this was not within the scope of this work (Figure S2). Although overall DO fluctuations were observed due to modified control cascades (Figure S3), 50% DO was not maintained throughout the cultivation. In particular, increasing the working volume led to fluctuations after 169 h. In addition, a technical defect and a blocked exhaust cooler reduced the oxygen input to avoid high pressure inside the bioreactor after 152 and 285 h. An additional filter heater was used to overcome this technical problem. Further optimizations can be made to improve the DO. For instance, including the control of the stirrer speed in the DO cascade could help to maintain a stable level. Furthermore, culturing at a higher working volume to increase the volume‐to‐surface ratio could further improve HCD cultivations in the MemStir system. After 288 h, no further changes to the DO cascade were made, and the perfusion was stopped after 300 h. Gassing was limited to 2000 cm^3^/min causing a DO drop to 10%.

While neither oxygen nor metabolites were limiting until perfusion was paused and the control of the DO cascade was not modified, even higher VCCs would most likely have led to a different limitation: viscosity (Clincke et al. 2013). The higher the VCC, the more difficult it was to measure the VCC of HEK‐LTV cells using the ViCell. Despite incubating the HEK‐LTV cells with trypsin and diluting them for VCC measurements, the viscosity of the samples prevented pipetting.

Previous publications regarding perfusion mode cultivations in STR reported maximum HEK cell concentrations of 93 × 10^6^ cells/mL and 111 × 10^6^ cells/mL (Schwarz et al. 2020; Kyung et al. 1994). Using suspension HEK293 cells and a hollow fiber system for cell retention, Kyung et al. observed that maintaining pH became critical at HCD, since increased gassing rates for CO_2_ stripping resulted in excessive foam formation (Kyung et al. 1994). Similar challenges were observed in the cultivation of CHO cells in perfusion mode. Schwarz et al. demonstrated that the process performance was altered at a VCC of > 80 × 10^6^ cells/mL (Schwarz et al. 2020). One of the main challenges was the gas exchange, resulting in an insufficient oxygen supply and impaired CO_2_ removal for pH control. Furthermore, excessive foam formation posed a risk of filter blockage and limited the VCC and viability of the process. In addition, antifoam agents, which are used to reduce foam formation, can impede the process. Accumulation of antifoam on the filter can lead to fouling and negatively impact cell viability and cell growth (Madabhushi et al. 2025; Velugula‐Yellela et al. 2018). These problems require intensive and time‐consuming process optimization studies when working with STRs. However, we did not observe any foam formation in our process, and efficient gas exchange enabled maintaining DO and pH set points at HCD most of the time (Figure S3, except for changes in control/setup). As previously mentioned, the measurement of maximum cell concentrations may have been affected by high viscosity, resulting in lower values than the actual concentration inside the reactor. Ozturk has described a 2‐ to 3‐fold increase in viscosity for cell concentrations above 50 × 10^6^ cells/mL (Ozturk 1996). High viscosity can be caused by an increased amount of biomass or by free DNA of lysed cells, for example (Elkin et al. 2015). Cultivating cells in a high‐viscosity environment can be challenging due to a reduction in k_L_a and increased transmembrane pressure associated with membrane‐based cell retention devices, such as hollow fibres (Clincke et al. 2013; Zhang et al. 2025). Additionally, the culture medium impacts the viscosity changes in metabolite concentrations, added growth factors and viscosity modifiers, such as dextran (Poon 2022). Accordingly, further optimization of the perfusion medium may allow to reduce the overall viscosity and thereby enabling even higher cell concentrations.

Finally, the choice of a specific bioreactor setup, including stirring and aeration systems, depends not only on maximum cell concentrations and maximum product titers achieved, but also on practicability, ease of use, and options for cleaning and reusing equipment. This is particularly relevant for large‐scale operation. Compared to conventional STRs with conventional stirrer and sparger systems (e.g., micro‐spargers or porous spargers using sintered metals or ceramics), the membrane‐stirrer technology is single‐use only. Therefore, the pros and cons of the technologies must be carefully evaluated considering aspects such as the cell concentrations to be achieved, the specific properties of the production cell line (i.e., shear sensitivity), medium properties, the risk of foaming as well as foam‐volume displacement, and the simplicity of process control and maintenance of the equipment.

Besides production processes operated at HCD, the MemStir system is highly relevant for the intensified cultivation of shear‐sensitive cells, such as human induced pluripotent stem cells, or natural killer cells. It enables the cultivation and differentiation of the cultivated cells by carefully controlling the shear forces (von Werz et al. 2026; Budeus et al. 2025). Currently, the system is only available up to 10 L working volume, but pilot scale devices (200 L) are in development and are of interest for the commercially cultivation of host cells up to high VCC to enable an intensified production. We believe this system could be an interesting alternative for intensified HCD production of viral vectors as vaccines or for oncolytic virotherapies. There, our research focuses on process intensification to enable high input doses and reduce the residence time of the temperature‐sensitive viruses (Jacobtorweihe et al. 2026; Göbel et al. 2026).

Overall, the MemStir system enabled HCD cultivation in perfusion mode with an ATF system, achieving a VCC of up to 93 × 10^6^ cells/mL at a cell‐specific growth rate of 0.022 h^−1^. The setup mitigated oxygen limitations and prevented foam formation.

## 4 Concluding Remarks

Efficient and sustainable production processes for recombinant proteins, viruses and viral vectors require intensified HCD cultivations. The MemStir system enabled an efficient, bubble‐free gas transfer (k_L_a 10–30 h^−1^) with comparable batch growth of HEK‐LTV cells compared to conventional STR and shake‐flasks. Culturing HEK‐LTV cells in perfusion mode using the MemStir system allowed for VCCs of 93 × 10^6^ cells/mL without foam formation or difficulties with CO_2_ stripping. Further studies should be performed to evaluate its performance across cell lines and scales, as well as for the actual production of, e.g., viruses or viral vectors with suitable product quality assessments. Overall, findings suggest that the MemStir system could be a beneficial alternative bioreactor system for intensified cell cultivation.

## Author Contributions

Conceptualization: Lennart Jacobtorweihe, Yasemin van Heuvel, Patrick Bongartz, Yvonne Genzel, Udo Reichl. Methodology: Lennart Jacobtorweihe, Yasemin van Heuvel. Investigation: Lennart Jacobtorweihe, Kathrina Scheiermann. Writing – original draft: Lennart Jacobtorweihe, Yasemin van Heuvel. Writing – review and editing: Lennart Jacobtorweihe, Yasemin van Heuvel, Kathrina Scheiermann, Patrick Bongartz, Yvonne Genzel, Udo Reichl. Supervision: Yasemin van Heuvel, Patrick Bongartz, Yvonne Genzel, Udo Reichl. Project Administration: Lennart Jacobtorweihe, Yasemin van Heuvel, Patrick Bongartz, Yvonne Genzel, Udo Reichl.

## Funding

The authors have nothing to report.

## Conflicts of Interest

P.B. declares that he is the inventor of the related patents “Integral gas‐introduction and stirring unit for gas‐liquid reactors”, RWTH Aachen University, 2021, WO2021152128 A1 and “Coupling element for coupling a drive element to a mixing unit for a fluid container, system and use”, RWTH Aachen University, 2024, WO2024240589A1. P.B. and Y.v.H. are employees of BioThrust GmbH, including Stock ownership and Stock options of it. This does not alter the authors' adherence to the policies of the journal. All other authors declare no personal or institutional conflicts of interest.

## Supporting information


Supporting File 1

Supporting File 2

Supporting File 3

Supporting File 4

Supporting File 5

Supporting File 6

## Data Availability

Data are available in the article and the Supporting materials. Additional data are available upon reasonable request from the authors. The data that support the findings of this study are available in the Supporting material of this article.
