# Supplementary material for: High Cell Density Cultivation of HEK293 Cells Using a 2L Membrane‐Stirred Bioreactor
Source: Biotechnol Bioeng. 2026 Jul 26;123(11):3165–74. doi: 10.1002/bit.70317 (PMC13665594; doi:10.1002/bit.70317)
Supplement: Supplementary file 1 [file BIT-123-3165-s005.docx]

**Supplementary**

**Figure S1: Characterization of k_L_a values at various aeration rates for the IKA habitat bioreactor, equipped with the MemStir system.** PBS, with working volumes of 0.6 L (■), 0.9 L (●) and 1.4 L (▲) and a fixed stirring speed of 60 rpm.

| **A**   | **B**   |
| --- | --- |

**Figure S2: Perfusion rates for the high-cell-density cultivations of HEK-LTV cells using the MemStir system.** **A** Cultivation with a fixed maximum total perfusion volume of 10.4 L. **B** Cultivation with a fixed maximum total perfusion volume of 28.2 L. Black line: perfusion rate, blue symbols: cell-specific perfusion rate (CSPR).

**Table S1:** Setting of the aeration cascade for HCD cultivations in perfusion mode. **A** Cascade for the first cultivation in perfusion mode. **B** Optimized cascade for the second cultivation in perfusion mode.

**A**

| Output [%] | Air [cc/min] | O_2_ [cc/min] | N_2_ [cc/min] |
| --- | --- | --- | --- |
| 0 | 100 | 20 | 80 |
| 20 | 200 | 50 | 0 |
| 40 | 200 | 100 | 0 |
| 60 | 100 | 200 | 0 |
| 80 | 0 | 300 | 0 |
| 100 | 0 | 400 | 0 |

**B**

| Output [%] | Air [cc/min] | O_2_ [cc/min] | N_2_ [cc/min] |
| --- | --- | --- | --- |
| 0 | 100 | 20 | 0 |
| 20 | 200 | 50 | 0 |
| 40 | 200 | 100 | 0 |
| 60 | 200 | 200 | 0 |
| 80 | 200 | 400 | 0 |
| 100 | 0 | 1500 | 0 |

| **A** | **B** |
| --- | --- |
|  |  |

**Figure S3:** Process parameters (stirrer speed, DO, temperature and pH) of the HCD cultivations in perfusion mode. **A** First cultivation. **B** second cultivation.
